# Involvement in decisions about intravenous treatment for nursing home patients: nursing homes versus hospital wards

**DOI:** 10.1186/s12910-018-0258-5

**Published:** 2018-05-08

**Authors:** Kristin Klomstad, Reidar Pedersen, Reidun Førde, Maria Romøren

**Affiliations:** 1 Centre for Medical Ethics, Institute of Health and Society, Faculty of Medicine, University of Oslo, P.O box 1130 Blindern, 0318 Oslo, Norway; 2 Antibiotic centre for primary care, Department of General Practice, Institute of Health and Society, Faculty of Medicine, University of Oslo, P.O box 1130 Blindern, 0318 Oslo, Norway

**Keywords:** Nursing home, Hospital, Elderly, End-of-life decisions, Life prolonging treatment, Advance care planning, Decision making capacity, Patient autonomy, Next of kin, Ethics

## Abstract

**Background:**

Many of the elderly in nursing homes are very ill and have a reduced quality of life. Life expectancy is often hard to predict. Decisions about life-prolonging treatment should be based on a professional assessment of the patient’s best interest, assessment of capacity to consent, and on the patient’s own wishes. The purpose of this study was to investigate and compare how these types of decisions were made in nursing homes and in hospital wards.

**Methods:**

Using a questionnaire, we studied the decision-making process for 299 nursing home patients who were treated for dehydration using intravenous fluids, or for bacterial infections using intravenous antibiotics. We compared the 215 (72%) patients treated in nursing homes to the 84 (28%) nursing home patients treated in the hospital.

**Results:**

The patients’ capacity to consent was considered prior to treatment in 197 (92%) of the patients treated in nursing homes and 56 (67%) of the patients treated in hospitals (*p* < 0.001). The answers indicate that capacity to consent can be difficult to assess. Patients that were considered capable to consent, were more often involved in the decision-making in nursing homes than in hospital (90% vs. 52%). Next of kin and other health personnel were also more rarely involved when the nursing home patient was treated in hospital. Whether advance care planning had been carried out, was more often unknown in the hospital (69% vs. 17% in nursing homes). Hospital doctors expressed more doubt about the decision to admit the patient to the hospital than about the treatment itself.

**Conclusions:**

This study indicates a potential for improvement in decision-making processes in general, and in particular when nursing home patients are treated in a hospital ward. The findings corroborate that nursing home patients should be treated locally if adequate health care and treatment is available. The communication between the different levels of health care when hospitalization is necessary, must be better.

**Trial registration:**

ClinicalTrials.gov NCT01023763 (12/1/09) [The registration was delayed one month after study onset due to practical reasons].

**Electronic supplementary material:**

The online version of this article (10.1186/s12910-018-0258-5) contains supplementary material, which is available to authorized users.

## Background

Decisions about life-prolonging treatment should be based on a clinical evaluation of the patient’s best interest – and on the patient’s own wishes [1]. Ethics and law should secure sound decision-making processes and that the patient’s voice is heard, also regarding the right to abstain from treatment.

Approximately 40,000 elderly people live in nursing homes in the last stage of their lives in Norway. About 80% suffer from cognitive impairments of different kinds and degrees. Nursing home patients live with an average of 5–7 diagnoses [2].

Nearly half of all deaths in Norway occur in residential care facilities [3]. A permanent resident in a Norwegian nursing home lives there for an average of 1.5–2 years before they die, but there are large variations [4]. When a nursing home patient suddenly gets worse, it is necessary to determine whether or not to give the patient life-prolonging treatment or palliative care, and whether or not to admit to hospital. Life-prolonging treatment is defined as all treatment and interventions that can delay a patient’s death [1]. Examples of this in nursing homes are intravenous fluids and antibiotic treatment.

When a patient no longer can express his or her own opinions, for instance due to cognitive impairment or confusion resulting from acute illness, the need to secure sound decision-making processes is even greater. When the patient lacks the capacity to consent, Norwegian health law demands more involvement of next of kin, and that other qualified health personnel are consulted [5]. The next of kin should be asked about their knowledge of what the patient would have wished if competent, for example previously expressed wishes on future treatment. It is important to clarify the patient’s preferences and values related to the intensity of life-prolonging treatment before it is too late, for instance through advance care planning (ACP) [1]. Wishes expressed in ACP or advance directives are not legally binding in Norway, but when the patient lacks capacity to consent the professionals should only provide treatment that are in the patient’s best interest and when it is likely that the patient would have consented to it [5]. That is, relevant previously expressed wishes about limiting life-prolonging treatment should as main rule be expected [1]. According to Norwegian law, it is the responsible professional to assess the patient’s capacity to consent and to make the final decision in cases where the patient’s capacity to consent is lacking. The assessment of the patient’s capacity is by and large entrusted to the responsible professional, but should assessed in relation to the concrete decision that has to be made and in accordance with justifiable professional standards. In questions about medical or life prolonging treatment, the responsible professional will be a physician. The Norwegian health law on informed consent, assessment of capacity and involvement of next of kin went into effect in 2001. Similar laws have been passed in many other countries the last decades, and reflect ethical and political discussions on the balance between paternalism and autonomy. The increased emphasis on patient autonomy is also reflected professionals’ codes of ethics, for example in the Norwegian associations for physicians and nurses. Many countries have also passed laws on advance directives and durable power of attorney. This is not yet the case in Norway. Thus role of the next of kin in Norway is not to consent on behalf of the patient in situations where capacity to consent is lacking, but to rather to inform the decision that the responsible professional should make. Appropriate involvement of the next of kin and the patient of still presupposes that the patient’s capacity to consent is assessed.

Patients, next of kin and health care staff may have different opinions on treatment intensity, which in turn may complicate decision-making. The question of treatment intensity is especially pressing for very ill patients who have reduced quality of life, short life expectancy, and when there is an increased risk of side effects from the treatment. The risk of confusion and side effects will often increase when frail nursing home patients are sent to the hospital [6]. In order to ensure the right level of treatment and treatment intensity, medical and ethical competence is necessary for nursing home staff. In addition, sufficient physician presence in the nursing home and good cooperation between the levels of health care are needed.

Many nursing home residents need to be admitted to hospital in order to get intravenous fluids or antibiotics because the nursing home lacks the skills or capacity to give this sort of treatment. This study is a part of the 3IV study: a large clinical intervention trial where all nursing homes in one administrative district were trained to administer intravenous treatment [7]. Because decisions about intravenous fluids or antibiotics to nursing home patients bring up many ethical dilemmas, research on the decision-making processes became a subproject. Earlier studies [8–14] indicate suboptimal decision-making processes, but there has been relatively little research done on how decisions about life-prolonging treatment for nursing home patients are made. This study is, as far as we know, unique by containing a large number of concrete patient trajectories.

The purpose of this study was to elucidate the decision-making process when life-prolonging treatment was given, here in the form of intravenous fluids or antibiotics, to nursing home patients, and in particular to compare how these decisions are made in nursing homes and in hospitals. We especially wanted to know more about who is involved in the decision making, whether capacity to consent is considered, the influence of capacity evaluations on patient involvement, how often ACP is carried out, and whether there were doubts about the treatment.

## Methods

### Study setting

All 34 nursing homes in a county were invited to participate in the study. Four declined to participate, two because the nursing home managers perceived little need for intravenous treatment among their residents, two because they used the hospital in the neighboring county. The 30 participating nursing homes had 12–124 beds, from one to eight wards, and either only one kind of ward, or a combination of different kinds: for rehabilitation, short and long term care, palliative care and separate wards for dementia. The nursing homes had 1 to 6 doctors employed (mean 1.9, median 1.5). Seven of the nursing homes (including the two largest nursing homes) had nursing home doctors employed in full time or half time positions; 21 used general practitioners (GPs) working 20% in the nursing homes (the majority of these split their time into 40% presence at the nursing home and 60% availability for telephone consultations); two of the large nursing homes had a combination of the two. Mean man-years for nurses in the nursing homes was 14.1 (range 3.5–40.2), mean man-years for nursing assistants were 26.2 (range 5 to 105). All admittance to hospital included in this study was to inpatient wards in the Department of Medicine at the local hospital, which is the only hospital in this county.

### Study design

The study is based on a sub-set of data from the 3IV-study [7], a using a modified stepped wedge cluster randomized trial with randomization on the nursing home level, each nursing home representing one cluster [15]. The intervention, a structured training program in intravenous treatment of dehydration and infections in nursing homes, was carried out in the 30 nursing homes following the randomization plan, from November of 2009 to December of 2011, with patient inclusion and data collection in the same period.

In nursing homes that had not received the training, the patients were admitted to the hospital for intravenous treatment. In nursing homes that had received the training, the patients were treated locally, given the staff had satisfactory skills and capacity, otherwise, they were hospitalized. Some of the nursing homes had the capacity to provide intravenous treatment before the project started.

### Study population

We included patients that were treated with intravenous fluids and/or intravenous antibiotics for pneumonia, upper urinary infections, or deep skin infections, either at the nursing home or at the hospital. Patients receiving intravenous antibiotics were defined as “intravenous antibiotics-patients” whether they were receiving intravenous fluids in addition, or not. Patients who were only receiving intravenous fluids were defined as “intravenous fluid-patients,” though several of them were also given per oral antibiotics. Patients with sepsis or patients who were admitted for accessory symptoms like anemia or other co-morbidity, were not included in the study. The total material of the 3IV-study consists of 330 patients; 108 patients were provided intravenous treatment in the hospital, 222 patients were provided treatment in the nursing homes. In this article, we present the results of the questionnaire about the decision-making process, filled out for 299 (91%) of the patients: 24 (22%) of the patients who were treated in the hospital, and 7 (3%) of those treated in the nursing home were excluded from the analysis due to incomplete answers.

### Data collection

In every nursing home, and on the wards of the Department of Medicine, there were one or two coordination nurses, who were responsible for inclusion of patients at the onset of intravenous treatment, and for registration of data in standardized questionnaires (Additional files 1, 2, 3, 4 and 5). Clinical data was registered on day one, and on specified days throughout the treatment course, such as diagnosis, vital signs (blood pressure, pulse, temperature, respiratory rate), and delirium (assessed using “Confusion Assessment Method (CAM)”. For the patients treated in the nursing homes, Barthel Index of Activities of Daily Living 14 days before disease onset was also recorded.

The focus of this paper is a separate section of the questionnaire that consisted of questions to the treating physician about the decision-making process before initiation of intravenous treatment: assessment of capacity to consent, involvement of patient, next of kin or other health personnel, and ACP. We also asked whether they had doubts about the treatment, and if so, reasons for their doubt. The hospital doctors were also asked whether they were in doubt that hospital admission had been the right course of action. The specific questions are listed in Table 2. It was the attending physician, a dedicated study nurse, or a nurse involved in the treatment of the patient that filled out the forms. The questions were developed based on earlier studies and questionnaires, and created to be as similar as possible for both the hospital and nursing home setting. The response alternatives were “Yes”, “No” and “I don’t know”.

### Analysis

IBM SPSS® statistics program version 22 was used for statistical analysis. For the purpose of this study, the analysis was conducted as in cross-sectional studies. In the comparisons between nursing homes and hospitals, and between nursing home doctors and GPs with part time positions, we used chi-squared test with significance level *p* < 0.05; Fisher’s exact test (two-sided) when expected cell count was < 5. Answers to open questions in the questionnaire were analyzed qualitatively using qualitative content analysis [16].

## Results

Of the 299 included patients, 215 (72%) received intravenous treatment in the nursing home; of these, 107 (50%) were treated with intravenous antibiotics. Among the 84 patients who received intravenous treatment in the hospital, 66 (79%) received intravenous antibiotics. Age, gender and course of disease among the patients who received treatment in the nursing homes and patients treated in hospital are presented in Table 1. Patient characteristics for the patients included in the 3IV-study are presented elsewhere [7].

**Table 1:** Characteristics and outcome in 299 patients provided intravenous antibiotics or fluids in nursing homes and hospital

	Nursing home *n* = 215 (%)	Hospital *n* = 84 (%)	*P*-values
Mean age (range)	80.6 (45–99)	83.0 (38–98)	NS
Median age	83.0	85.0	NS
Women	123 (57%)	48 (57%)	NS
Provided intravenous antibiotics	107 (50%)	66 (79%)	< 0.001
Died within 30 days	66 (31%)	19 (23%)	NS

### Involving the patient, next of kin and other health professionals

Overall, the respondents in the nursing homes reported more often than the respondents in the hospital that the treatment was discussed with the patient (133 (62%) vs. 19 (23%), *p* < 0.001), next of kin (113 (53%) vs. 21 (25%), *p* < 0.001) and other health professionals who knew the patient (191 (89%) vs. 18 (21%), *p* < 0.001). The responses from the hospital were more often than from nursing homes “I don’t know.”

The patient’s capacity to consent was reported considered in 197 (92%) of the patients treated locally and in 56 (67%) of the patients treated in the hospital (*p* < 0.001). Among these 253 patients, the treatment was discussed with 126 (83%) of the patients who had the capacity to consent before treatment commenced, and with 14 (14%) of the patients without capacity to consent (*p* < 0.001). Patients with capacity to consent were more often involved in treatment decisions in the nursing homes: 112 (90%) of patients treated in the nursing homes; 14 (52%) of patients treated in the hospital (Fisher’s exact test < 0.001 (two-sided)) (Table 2).

**Table 2:** Questions that give insight into the decision-making process. Responses from nursing homes and hospital compared

Questions		Answers	Nursing homes *n* = 215(%)	Hospitals *n* = 84(%)	*P*-value or Fisher’s exact test^*^
Was the patient’s capacity to consent assessed before commencing treatment?		Yes	197(92)	56(67)	*p* < 0.001
		No	12(6)	5(6)	
		I don’t know	6(3)	23(27)	
If yes, did the patient have the capacity to consent?		Yes	124(63)	27(48)	*p* < 0.05
		No	73(37)	29(52)	
Was the treatment discussed with the patient before commencement?	Capacity to consent *n* = 124 in nursing home *n* = 27 in hospital	Yes	112(90)	14(52)	Exact< 0.001
		No	9(7)	4(15)	
		I don’t know	3(2)	9(33)	
	Not capacity to consent *n* = 73 in nursing home *n* = 29 in hospital	Yes	14(19)	0(0)	Exact< 0.01
		No	59(81)	28(97)	
		I don’t know	0(0)	1(3)	
Was the treatment discussed with the next of kin before commencement?	Capacity to consent	Yes	59(48)	8(30)	Exact< 0.01
		No	58(47)	10(37)	
		I don’t know	7(6)	9(33)	
	Not capacity to consent	Yes	47(64)	3(10)	Exact< 0.001
		No	22(30)	12(41)	
		I don’t know	4(6)	14(48)	
Was the treatment discussed with health personnel who knew the patient?	Capacity to consent	Yes	115(93)	8(30)	Exact< 0.001
		No	8(7)	6(22)	
		I don’t know	1(1)	13(48)	
	Not capacity to consent	Yes	62(85)	4(14)	Exact< 0.001
		No	7(10)	11(38)	
		I don’t know	4(6)	14(48)	
Advance care planning done in the past?^a^		Yes	108(50)	14(17)	*p* < 0.001
		No	71(33)	12(14)	
		I don’t know	36(17)	58(69)	

^*^Less than 5 patients counted in one or more cells

^a^Full question: Have there *previously* been carried out conversations with the patient or next of kin regarding the patient’s wishes and values relating to life-prolonging treatment and what to do if the patient suddenly gets much worse?

There was no significant difference in discussing treatment with next of kin in the patients with and without capacity to consent (67 (44%) vs. 50 (49%), *p* = NS). Among the patients lacking capacity to consent, treatment was discussed with next of kin more often in the nursing homes than in the hospital (47 (64%) vs. 3 (10%), Fisher’s exact test < 0.001 (two-sided)) (Table 2).

The treatment was more often discussed with other health professionals in patients with, compared to patients without, capacity to consent (123 (82%) vs. 66 (65%), *p* < 0.05). Discussions among other health personnel were more common in the nursing homes than in the hospital, regarding both patients with and without capacity to consent (Table 2).

The reasons given for not discussing the treatment with patients who had the capacity to consent, and the reasons given for not discussing treatment with the next of kin to patients without capacity to consent are summed up in Table 3.

**Table 3:** Reasons given for not discussing treatment with patients or next of kin dependant of capacity to consent. Responses from both nursing homes and hospitals

Reasons for not discussing treatment with patients that have the capacity to consent
Medical
Admission was necessary
Patient was somnolent
Patient suddenly got worse and was not possible to get through to /communicate with
Patient does not communicate well due to reduced speech
Practical
Commenced treatment after telephone conversation with patient’s doctor
The doctor did not have time
Treatment was discussed with next of kin
Reasons for not discussing treatment with next of kin of patient’s who lack the capacity to consent:
Medical
All treatment should be tried
We had to start treatment. We informed them afterwards
Obviously necessary and correct to start treatment
Practical
Next of kin not easily contacted
Patient did not have close next of kin
Treatment was prescribed by physician by telephone

### Advance care planning (ACP)

It was reported that ACP had been carried out in 108 (50%) of the patients treated locally versus in 14 (17%) of the admitted patients (*p* < 0.001) (Table 2). The hospital responded “I don’t know” in 58 (69%) of the cases.

### Doubt

The respondents reported doubt about whether intravenous treatment was right for 69 (23%) of the patients. In 45 (15%) of the patients, doubt was reported about whether the treatment would have an effect; in 38 (13%) whether the treatment was in the patient’s best interest; in 25 (8%), doubt about the patient’s preferences. There were not significant differences in doubt in the nursing homes compared to the hospital (55 of 215 (26%) vs. 14 of 84 (17%), *P* = 0.1). Further, there were not significant differences in doubt in cases where the treatment had been discussed with the patients, compared with the cases where the patient was not involved (31 of 152 (20%) vs. 32 of 111 (29%), *p* = 0.11). There was more doubt reported in cases where treatment was discussed with next of kin than when next of kin was not involved (40 of 134 (30%) vs. 21 of 112 (19%), *p* < 0.05. There was no correlation between reported doubt and involvement of other health personnel. There was no doubt about treatment reported in the 34 patients treated in nursing homes with a Barthel Index score of > 11 of 20, and there was less doubt about the 101 patients who after 30 days returned to their former level of functioning, than the 196 patients with reduced health status or death (16% vs. 27%, *p* > 0.05).

The hospital respondents reported doubt about whether the admission was right for 30 (36%) of the 84 patients admitted. Among the patients treated at the hospital, reasons for doubt were: The patient could have been treated in the nursing home (21 (25%)); uncertainty about whether the benefits of admission outweighed the disadvantages (11 (13%)), and doubts about whether life-prolonging treatment was right for the patient (9 (11%)).

### The influence of the nursing home doctors’ position

The influence of the nursing home doctors’ position on the decision process was explored among patients treated in the nursing homes (Table 4). For patients from nursing homes with full or half-time nursing home doctors, it was reported that the treatment was discussed with the patients more often than for patients from nursing homes with GPs with 20% positions. Oppositely, for patients from nursing homes with GPs, it was more often reported that the treatment was discussed with the next-of-kin. We did not find significant differences in reported ACP, or doubt about treatment.

**Table 4:** Questions that give insight into the decision-making process for patients treated in nursing homes with nursing home doctors on staff, versus in nursing homes with general practitioners (GPs) in 20% positions

	Patients treated in nursing homes with full- or part time physicians *n* = 125	Patients treated in nursing homes with GPs *n* = 68	*P*-value
The patient’s capacity to consent was assessed	112 (92%)	60 (91%)	NS
The treatment was discussed with the patient before commencement	82 (67%)	31 (47%)	< 0.05
The treatment was discussed with the next of kin before commencement	54 (44%)	41 (62%)	< 0.05
Was the treatment discussed with health personnel who knew the patient?	109 (89%)	60 (91%)	NS
Advance care planning done in the past^a^	67 (55%)	30 (46%)	NS
Doubt about whether intravenous treatment was the right course of action for this patient	27 (22%)	20 (30%)	NS

^a^Full question: Have there *previously* been carried out conversations with the patient or next of kin regarding the patient’s wishes and values relating to life-prolonging treatment and what to do if the patient suddenly gets much worse?

## Discussion

This study shows that nursing home patients are, at least sometimes, given intravenous treatment without the patient, next of kin or other health personnel being involved in the decision-making process, as the law and ethical guidelines mandate they should be. There is also a relatively large proportion of patients who have not been given the opportunity for ACP [17].

Due to demographic changes and an intensified effort in community care of the elderly [18], residents in European nursing homes have over the past decades become increasingly frail, often with multiple active diagnoses [19], and the situation is similar in Norway. The quality, quantity, and nature of care in nursing homes varies across nations [20, 21]. Nevertheless, they share a common need for solid and legal decisions regarding health interventions for nursing home patients.

Quality of care in nursing homes is considered a complex and multidimensional phenomenon, influenced by resident, staffing and ward characteristics [22]. International studies indicate that, for the most part, higher total staffing levels and higher educational level among staff are positively associated with quality of care [23]. Norwegian nursing homes have high levels of nurses and physicians compared to many other countries [20, 24], and are thus in a position to emphasize and improve both medical and ethical aspects of life-prolonging treatment.

Involvement of the nursing home patient, next of kin, and other health professionals, as well as ACP and assessment of capacity to consent, more commonly follows legal and ethical guidelines in nursing homes than in the hospital, according to this study. Our findings suggest that the nursing homes that have a physician present most of the time, more often involve the patient in the decision-making processes. This coincides with an earlier qualitative study from Norway [17]. The responses also shows that when a nursing home patient is treated in the hospital, the professionals know less about how the different parties have been involved, whether the patient’s capacity to consent has been assessed, and whether or not ACP has been carried out. This can mean that even if ACP was carried out in the nursing home, the relevant information does not follow the patient when admitted to the hospital. Or, that if such conversations were carried out earlier in the hospital during this or previous admissions, the information is not easily accessible to use as relevant support in the decision-making processes.

From earlier studies, we know that many health professionals find it hard to assess capacity to consent [25]. In our study, it seems that capacity has been assessed for a large majority of the patients. The answers to why patients who have the capacity to consent are not involved in treatment decisions, for instance that “Patient suddenly got worse and was not possible to get through to/communicate with” indicate, however, that some of the capacity assessments are deficient. A possible reason for this is that training in how to assess capacity has been given limited attention in medical training in Norway.

The patient’s capacity to consent seems to have little impact on whether next of kin and other health professionals are involved, in contrast to what the law and ethical guidelines mandate. If we see that in connection with the fact that patients and next of kin are rarely included, and the reasons given for not including them, one possible interpretation is that the medical assessment of what is necessary is what steers the treatment of nursing home patients. The patient’s individual preferences have a more peripheral role, and are in many cases left out of decision-making regarding intravenous treatment.

In this study, a relatively large proportion (28% in total) of the patients died within 30 days, while doubt about intravenous treatment being right was more rarely reported (23% in total). One may ask whether doubt – in the treatment of very frail and sick, and sometimes dying, nursing home patients - is expressed or admitted too rarely. We know that both under and over-treatment are problems in health care for the elderly [26]. In this study, we have only included situations where the patient received treatment. However, the topics we have studied – e.g. doubt - are equally relevant in situations where treatment is withheld or withdrawn.

In the hospital, there was more often doubt about admissions than about the treatment itself. This may mean that they feel that the patient should be treated, but at a lower level, and that once a patient is admitted, treatment is the right course of action. Combining this with our findings that imply a lesser degree of inclusion of involved parties in decision-making processes, and challenges regarding the flow of information between the levels of health care [27], this is an important reason to treat as many patients as possible in the nursing home, if the nursing home is able to provide diagnostics and treatment of similar quality to the hospitals. The results from the 3IV study [7] show that the nursing homes are able to do this when more extensive or advanced diagnostics or treatment are not needed.

Our findings also reveal a need for better communication between the levels of health services, development of better documentation systems, routines and competence regarding capacity assessment - and the use of such assessments, ACP, and finally, a need for better inclusion of patient, next of kin and other health personnel in decision making regarding life-prolonging treatment in general.

### Strengths and weaknesses

A decision-making process is complex and consists of clinical aspects in addition to the formal and legal guidelines for how to do it. A questionnaire is suited to map out a situation, but can result in over-reporting because the respondents intuitively know what the “right” answer is. A high number reporting ACP in this study, compared to other studies, may indicate this kind of bias [13, 28]. An earlier study carried out by von Hofacker et al. pulled information from patient charts [29]. Using that method may result in under-reporting.

Not all questionnaires were answered by the treating physician as we requested, but instead by a nurse. However, nurses are often precise, and access to the physician chart was necessary in order to answer other questions in the study. In those cases where a nurse answered the questionnaire, we called afterwards to talk to them. They usually said that the questions wereanswered after consulting the doctor.

Using a questionnaire, you risk differing interpretations of questions. “To discuss” is a phrase that may be associated with different things, such as disagreement or conflict. Yet, since so many have responded in the positive to the question of discussing with the patient prior to treatment, we can assume that most of them have understood this to mean a conversation with the patient.

Although we through the inclusion criteria aimed to ensure comparability between the patients treated in nursing homes and the patients admitted to hospital, the two groups are not identical. We assume that in the study as well as in clinical practice, there is a trend towards more seriously ill patients being hospitalized [7]. However, among patients given intravenous treatment locally, there will be some that are provided intravenous treatment as palliative care in a terminal phase who would not been hospitalized for the same treatment. How this affects the decision processes and thereby the outcomes of the study is difficult to assess. A second difference between the groups is that among the patients treated in the hospital decision about hospitalization has already been made – which may lead the hospital doctors to think that a decision to provide life-prolonging treatment has already been made. This is often not true; reasons for hospitalization of these patients are multifactorial and not necessarily based on the patients will or what is best for the patient [29]. In this study, hospital doctors expressed more doubt about the decision to admit the patient to the hospital than about the treatment itself. In a qualitative sub-study of the 3IV project we showed that both nursing home- and hospital doctors were concerned by unnecessary hospitalizations and overtreatment in the hospital [30]. Thus, ethically and legally sound decision processes are equally important when providing treatment to the elderly patients in the hospital as in the nursing home.

An advantage with our study design is that we get comparable data from many concrete patient treatment trajectories, and many different types of nursing homes, nearly all in one administrative district. The 3IV study also contains a number of clinical as well as qualitative data [7, 30, 31], that have been used as assistance in the interpretation of the data from the questionnaires about decision making.

The current study included the vast majority of public and private nursing homes in one, relatively big county, i.e. the whole spectrum of resident and ward characteristics from both rural and urban areas. The number of physician hours per resident per week in the county was 0.62 in 2016, the mean for Norway was 0.55 (0.38 to 0.75) [24]. Although we cannot claim that the presented results are fully representative for the situation in Norway, the nursing homes in this study are probably without major differences from Norwegian nursing homes in general.

## Conclusions

In our study we find better decision-making processes and access to information relevant do such decisions - for example the patient’s preferences and assessments of capacity to consent - in nursing homes than in hospitals. The results point to a potential for better involvement of nursing home patients and their next of kin before decisions are made about life-prolonging treatment both in hospitals and nursing homes. Our findings also indicate that the patient’s capacity to consent is not always considered, and that ACP is often not carried out. Patient preferences expressed through ACP can be difficult to interpret when a situation arises. Still, it is important that capacity to consent is assessed, that patients are involved when able to consent, and that a decision about life-prolonging treatment is made by a doctor in collaboration with someone who knows the patient well, if the patient lacks the capacity to consent. Only then can the professionals decide whether the health intervention is in line with the patient’s interest. Determining the right thing to do for a severely ill nursing home patient certainly requires biomedical expertise, but it is also to a large degree a value question, where the patient’s wishes and values need to be central [1]. Adequate decision making processes probably requires sufficient training, appropriate routines and documentation systems, and that the professionals have time for involvement of patient and next of kin, documentation, and to discuss the ethical dilemmas that arises.

## Additional files

Additional file 1: Research form. Basic form for all patients. (DOC 82 kb)

Additional file 2: Research form. Form for patients treated with iv antibiotics in hospital. (DOC 103 kb)

Additional file 3: Research form. Form for patients treated with iv antibiotics in nursing homes. (DOC 88 kb)

Additional file 4: Research form. Form for patients treated with iv fluids in hospital. (DOC 74 kb)

Additional file 5: Research form. Form for patients treated with iv fluids in nursing homes. (DOC 62 kb)

## Abbreviations

ACP: Advanced care planning
GP: General practitioner
NS: Not significant
vs: Versus
