# Supplementary material for: Involvement in decisions about intravenous treatment for nursing home patients: nursing homes versus hospital wards
Source: BMC Med Ethics. 2018 May 8;19:34. doi: 10.1186/s12910-018-0258-5 (PMC5941318; doi:10.1186/s12910-018-0258-5)
Supplement: Supplementary file 1 — Research form. Basic form for all patients. (DOC 82 kb) [file 12910_2018_258_MOESM1_ESM.doc]

| Name ____________________D.o.b. ____________ | | **Form 1 – Common patient information, p1** | |
| --- | --- | --- | --- |
| Sex  F  M | |  | |
| Nursing home ___________________________________ | Type of ward  Rehabilitation   Short term ward   Long term ward   Combined short and long term   Dementia / closed ward   Palliative ward | |  |

| First examination NURSE | Date  (ddmmyy) |
| --- | --- |

| **New unspecified symptoms / findings** | | **Urinary tract**  Uncomfortable/frequent urination  New/increased urinary incontinence/retention  Change of color/smell or cloudy urine  Urine stix  *Leukocytes____ Nitrite____ Blood___*  **Respiratory**  Gurgling respiration  Cough  **Skin/tissue**  Wounds with pus/incrustation  Redness  Heat  Swelling  Localised pain  **Dehydration**  Dry skin  Poor skin elasticity  Reduced urine production  Blood samples  *Hgb ___.__ B-glucose___.__* |  No  Yes   No  Yes   No  Yes   No  Yes   No  Yes   No  Yes   No  Yes   No  Yes   No  Yes   No  Yes   No  Yes   No  Yes   No  Yes   No Yes   No  Yes   No Yes |
| --- | --- | --- | --- |
| Fever  Fever with chills  Reduced general condition  Uncomfortable/gurgling respiration  Nausea/emesis  Stomach and/or back pain  Falling down/tendency to fall  Other_________________________  ______________________________ |  No  Yes   No  Yes   No  Yes   No  Yes   No  Yes   No  Yes   No  Yes   No  Yes |

Clinical status day 1

| BP ____/____ Pulse _____ Temp ___.__ Respiration frequency____ CRP ____  Consciousness  Awake  Somnolence  Unconscious  Food intake  Normal  Reduced  Probe  Liquid intake  Normal  Reduced  Intravenous fluid |
| --- |

|  Confusion Assessment Method (CAM) completed (p 4)   Barthel ADL-Index completed (p 5)   Copy of medication list attached | |
| --- | --- |
| Name:________________________D.o.b.:____________ | **Form 1 – Common patient information, p2** |

| Conclusion first examination PHYSICIAN | Date  (ddmmyy) |
| --- | --- |

| **Type of consultation** | 1.  2. |  Emergency room doctor   Telephone consultation |  Nursing home doctor   Clinical examination |
| --- | --- | --- | --- |

| **Tentative diagnosis**  (one/several) |  Dehydration   Upper respiratory infection   Lower respiratory infection   Deteriorating COPD or chronic bronchitis   Lower urinary tract infection   Upper urinary tract infection   Surface infection on skin, infected diabetic foot sore etc   Deep infection on skin   Post-operative wound infection, surface   Post-operative wound infection, deep   Other: ______________________________________________________   Unsure: |
| --- | --- |

Measures:

 Only palliative treatment in the nursing home (not antibiotics or intravenous fluids)

 Treatment in hospital

|  Peroral antibiotics | **Form 2 – PO/IM ANTIBIOTICS** |
| --- | --- |

|  Only intravenous fluids | **Form 3 – IV FLUIDS** |
| --- | --- |

|  Intravenous antibiotics | **Form 4 – IV ANTIBIOTICS** |
| --- | --- |

 Hospital admittance

|  Assumed need for IV treatment – nursing home lacks training   Assumed need for IV treatment – nursing home lacks capacity or competence right now  Explain further_______________________________________________________________________  _____________________________________________________________________________________  _____________________________________________________________________________________   Intravenous treatment in the nursing home is not relevant because of:  1.  Complicating factors/comorbidity  2.  Serious infection of the abdomen  3. ** Seriousness/sepsis development (admittance should be considered if three or more criteria are present): Temperature >38.5 or<35.0  Pulse >100/min  Respiration >30/min  Systolic BP < 90 mmHg  Oxygen saturation < 92%   Needs a diagnostic assessment, may come back for intravenous treatment |
| --- |

| Name:________________________D.o.b.:____________ | **Form 1 – Common patient information, p3** |
| --- | --- |

| Background information To be completed by nursing home doctor |
| --- |

| Known illness | |  |
| --- | --- | --- |
|  | COPD  Angina/previous myocardial infarction  Atrial fibrillation  Heart failure  Dementia  Cerebral stroke  Diabetes  Cancer disease (which type?):______________________________________  Foot/leg sore  Bed sore/pressure sore  Reduced peripheral circulation  Other:_____________________________________________________ |  No  Yes   No  Yes   No  Yes   No  Yes   No  Yes   No  Yes   No  Yes   No  Yes   No  Yes   No  Yes   No  Yes   No  Yes |
| Risk of urinary tract infection | |  |
|  | Urine incontinence, wears diapers  Intermittent urine catheterization  Permanent urine catheterization |  No  Yes   No  Yes   No  Yes |
| Risk of hospital-related infection | |  |
|  | From hospital < 48 h  Operated last 30 days  Operated in foreign substance last 12 mo |  No  Yes   No  Yes   No  Yes |

| **Day 1** Confusion Assessment Method (CAM) | **Form 1 – Common patient information, p4** |
| --- | --- |

| Inouye et al. Ann Int Med 1990; 113: 941-948.  Norwegian translation by Anette Hylen Ranhoff, Marianne Hjermstad and Jon Håvard Loge, 2004. |
| --- |

**IN GENERAL**

Delirium (also called acute deterioration or acute confusion) is a normal complication of acute illness in the elderly. There are several types, the patients may become either hyperactive (agitated), hypoactive (quiet), or go between both. The severity may vary significantly.

Delirium requires quick diagnosis and intervention. Confusion Assessment Method (CAM) is a short screening test that can diagnose with good precision (based on DSM-III and ICD-10). The algorithm is well suited for finding and treating delirium in clinical practice. The questions should be answered by health care personnel,, and based on their own knowledge of the patient, or conversations with staff or next of kin that know the patient.

**The Confusion Assessment Method Instrument**

**1. *[Acute Onset]*** Is there evidence of an acute change in mental status from the patient's baseline?


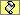
**2A. *[Inattention]*** Did the patient have difficulty focusing attention, for example, being easily distractible, or having difficulty keeping track of what was being said?

**2B. *(If present or abnormal)*** Did this behavior fluctuate during the interview, that is, tend to come and go or increase and decrease in severity?

**3. *[Disorganized thinking]*** Was the patient's thinking disorganized or incoherent, such as rambling or irrelevant conversation, unclear or illogical flow of ideas, or unpredictable switching from subject to subject?

**4. *[Altered level of consciousness]*** Overall, how would you rate this patient's level of consciousness? (Alert [normal]; Vigilant [hyperalert, overly sensitive to environmental stimuli, startled very easily], Lethargic [drowsy, easily aroused]; Stupor [difficult to arouse]; Coma; [unarousable]; Uncertain)

**5. *[Disorientation]*** Was the patient disoriented at any time during the interview, such as thinking that he or she was somewhere other than the hospital, using the wrong bed, or misjudging the time of day?

**6. *[Memory impairment]*** Did the patient demonstrate any memory problems during the interview, such as inability to remember events in the hospital or difficulty remembering instructions?

**7. *[Perceptual disturbances]*** Did the patient have any evidence of perceptual disturbances, for example, hallucinations, illusions or misinterpretations (such as thinking something was moving when it was not)?

**8A. *[Psychomotor agitation]*** At any time during the interview did the patient have an unusually increased level of motor activity such as restlessness, picking at bedclothes, tapping fingers or making frequent sudden changes of position?

**8B. *[Psychomotor retardation].*** At any time during the interview did the patient have an unusually decreased level of motor activity such as sluggishness, staring into space, staying in one position for a long time or moving very slowly?

**9. *[Altered sleep-wake cycle].*** Did the patient have evidence of disturbance of the sleep-wake cycle, such as excessive daytime sleepiness with insomnia at night?

| Barthel ADL-Indeks (status 14 days ago) | **Form 1 – Common patient information, p 5** |
| --- | --- |

| Mahoney FI, Barthel DW. Maryland State Med J 1965;14:61-65.  This Norwegian version was edited in 2008 by Ingvild Saltvedt, Jorunn L. Helbostad, Unni Sveen, Pernille Thingstad, Olav Sletvold  and Torgeir Bruun Wyller on the basis of several Norwegian translations, and with emphasis on the original publication from 1965. |
| --- |

**IN GENERAL**

Barthel ADL-index is first and foremost meant to be used by nurses, occupational therapists and physical therapists in their daily interaction with the patients. It should register what the patient actually does, not what you think the patient can handle. The answers should be based on knowledge of the patient, or conversations with staff or next of kin who know the patient. The patient should not be “tested”. The points represent degree of independence from help from another person, regardless of the reason for it. If supervision or adaptation is necessary, the person is not independent, but if an activity is possible by using aids, the person is independent.

*Bowels* (preceding week)

• If needs enema from nurse, then 'incontinent.'

• 'Occasional' = once a week.

*Bladder* (preceding week)

• 'Occasional' = less than once a day.

• A catheterized patient who can completely manage the catheter alone is registered as 'continent.'

*Grooming* (preceding 24 – 48 hours)

• Refers to personal hygiene: doing teeth, fitting false teeth, doing hair, shaving, washing face. Implements can

be provided by helper.

*Toilet use*

• Should be able to reach toilet/commode, undress sufficiently, clean self, dress, and leave.

• 'With help' = can wipe self and do some other of above.

*Feeding*

• Able to eat any normal food (not only soft food). Food cooked and served by others, but not cut up.

• 'Help' = food cut up, patient feeds self.

*Transfer*

• From bed to chair and back.

• 'Dependent' = NO sitting balance (unable to sit); two people to lift.

• 'Major help' = one strong/skilled, or two normal people. Can sit up.

• 'Minor help' = one person easily, OR needs any supervision for safety.

*Mobility*

• Refers to mobility about house or ward, indoors. May use aid. If in wheelchair, must negotiate corners/doors

unaided.

• 'Help' = by one untrained person, including supervision/moral support.

*Dressing*

• Should be able to select and put on all clothes, which may be adapted.

• 'Half' = help with buttons, zips, etc. (*check!*), but can put on some garments alone.

*Stairs*

• Must carry any walking aid used to be independent.

*Bathing*

• Usually the most difficult activity.

• Must get in and out unsupervised, and wash self.

• Independent in shower = 'independent' if unsupervised/unaided.
