# Supplementary material for: Involvement in decisions about intravenous treatment for nursing home patients: nursing homes versus hospital wards
Source: BMC Med Ethics. 2018 May 8;19:34. doi: 10.1186/s12910-018-0258-5 (PMC5941318; doi:10.1186/s12910-018-0258-5)
Supplement: Supplementary file 3 — Research form. Form for patients treated with iv antibiotics in nursing homes. (DOC 88 kb) [file 12910_2018_258_MOESM3_ESM.doc]

| Name:________________________D.o.b.:____________ | **NH form – IV ANTIBIOTICS p1** | | | |
| --- | --- | --- | --- | --- |
| Sex  F  M | | |  | |
|  | |  | |  |

| **Diagnosis**  (one/several) |  Pneumonia   Upper urinary tract infection |  Deep skin infection   Post-operative wound infection |  Unsure __________________   Other ____________________ |
| --- | --- | --- | --- |

Acute treatment

| Date | Time of first dose | Medication  (product name) | Daily dose  (mg/g/IE) | Dosage  (mg/g/IE)x__ | Date of cessation | Treatment failure? (yes/no) | Number of days treated |
| --- | --- | --- | --- | --- | --- | --- | --- |
|  |  |  |  |  |  |  |  |
|  |  |  |  |  |  |  |  |
|  |  |  |  |  |  |  |  |
|  |  |  |  |  |  |  |  |
|  |  |  |  |  |  |  |  |
|  |  |  |  |  |  |  |  |
|  |  |  |  |  |  |  |  |

Discontinued medication: ____________________________________________________________________

Other treatment initiated: __________________________________________________________________

_________________________________________________________________________________________

Clinical status day 1

| BP ____/____ Pulse _____ Temp ___.__ Respiration frequency____ CRP ____  Consciousness  Awake  Somnolence  Unconscious  Food intake  Normal  Reduced  Probe  Liquid intake  Normal  Reduced  Intravenous fluids |
| --- |

|  Form about decision-making is filled out (p 5)   Confusion Assessment Method (CAM) filled out for day 1   Copy of medication list day 1 attached |
| --- |

| Name:________________________D.o.b.:____________ | **NH form – IV ANTIBIOTICS p2** |
| --- | --- |

Clinical status day 2

| BP ____/____ Pulse _____ Temp ___.__ Respiration frequency____ CRP ____ (if taken)  Consciousness  Awake  Somnolence  Unconscious  *Fill out for those patients who had aberrant results on day 1* |
| --- |

|  Confusion Assessment Method (CAM) filled out for day 2 |
| --- |

Important notes (i.e. complications)______________________________________________________________

_________________________________________________________________________________________________

_________________________________________________________________________________________________

Clinical status day 3

| BP ____/____ Pulse _____ Temp ___.__ Respiration frequency____ CRP ____ (if taken)  Consciousness  Awake  Somnolence  Unconscious  *Fill out for those patients who had aberrant results on day 2* |
| --- |

|  Confusion Assessment Method (CAM) filled out for day 3 |
| --- |

Important notes (i.e. complications)______________________________________________________________

_________________________________________________________________________________________________

Clinical status day 4

| BP ____/____ Pulse _____ Temp ___.__ Respiration frequency____ CRP ____ (if taken)  Consciousness  Awake  Somnolence  Unconscious  *Fill out for those patients who had aberrant results on day 3* |
| --- |

|  Confusion Assessment Method (CAM) filled out for day 4 |
| --- |

Important notes (i.e. complications)______________________________________________________________

_________________________________________________________________________________________________

_________________________________________________________________________________________________

Clinical status day 5

| BP ____/____ Pulse _____ Temp ___.__ Respiration frequency____ CRP ____ (if taken)  Consciousness  Awake  Somnolence  Unconscious  *Fill out for those patients who had aberrant results on day 4* |
| --- |

|  Confusion Assessment Method (CAM) filled out for day 5 |
| --- |

Important notes (i.e. complications)______________________________________________________________

_________________________________________________________________________________________________

_________________________________________________________________________________________________

| Name:________________________D.o.b.:____________ | **NH form – IV ANTIBIOTICS p3** |
| --- | --- |

Clinical status day 7

| BP ____/____ Pulse _____ Temp ___.__ Respiration frequency____ CRP ____ (if taken)  Consciousness  Awake  Somnolence  Unconscious  *Fill out for those patients who had aberrant results on day 5* |
| --- |

|  Confusion Assessment Method (CAM) filled out for day 7 |
| --- |

Important notes (i.e. complications)______________________________________________________________

_________________________________________________________________________________________________

_________________________________________________________________________________________________

Clinical status day 10

| BP ____/____ Pulse _____ Temp ___.__ Respiration frequency____ CRP ____ (if taken)  Consciousness  Awake  Somnolence  Unconscious  *Fill out for those patients who had aberrant results on day 7* |
| --- |

|  Confusion Assessment Method (CAM) filled out for day 10 |
| --- |

Important notes (i.e. complications)______________________________________________________________

_________________________________________________________________________________________________

_________________________________________________________________________________________________

Clinical status day 15

| BP ____/____ Pulse _____ Temp ___.__ Respiration frequency____ CRP ____ (if taken)  Consciousness  Awake  Somnolence  Unconscious  *Fill out for those patients who had aberrant results on day 10* |
| --- |

|  Confusion Assessment Method (CAM) filled out for day 15 |
| --- |

Important notes (i.e. complications)______________________________________________________________

_________________________________________________________________________________________________

_________________________________________________________________________________________________

Clinical status day 30

| BP ____/____ Pulse _____ Temp ___.__ Respiration frequency____ CRP ____ (if taken)  Consciousness  Awake  Somnolence  Unconscious  *Fill out for those patients who had aberrant results on day 15* |
| --- |

|  Confusion Assessment Method (CAM) filled out for day 30 |
| --- |

Important notes (i.e. complications)______________________________________________________________

_________________________________________________________________________________________________

| Name:________________________D.o.b.:____________ | **NH form – IV ANTIBIOTICS p4** |
| --- | --- |

Summary 30 days after debut of current illness

| **Course of illness**  ___ days of intravenous fluid  ___ days before clinically well  Back to normal condition?  Yes  No  Do not know patient’s normal condition   Dead ___ days after treatment initiation | | |
| --- | --- | --- |
| **Complications**  Reaction to antibiotics    Bed sore  Fall with injury  Delirium  Hospital infection  Other |  No Yes   No Yes:   No Yes:   No Yes:   No Yes:   No Yes: |  Nausea/emesis  Rash   Shock  ________________________________________  ________________________________________  ___ days  ________________________________________  ________________________________________ |
| **Intravenous treatment**  Complications from intravenous treatment (infection in vein, hematoma, failure in equipment etc.)   No  Yes:_____________________________________________________________________________  Challenges/disadvantages of treating this patient in the nursing home:_________________________________  _________________________________________________________________________________________  _________________________________________________________________________________________  Advantages to treating this patient in the nursing home: ____________________________________________  _________________________________________________________________________________________  _________________________________________________________________________________________ | | |

Check list 30 days after debut of current illness

|  Consent form attached (patient’s signature of participation in the study)   Barthel ADL-Index filled out – for situation 30 days after debut of illness   Copy of current medication list attached   Patient is discharged to the home. Call: Lisbeth Østby, 91820728   Patient is dead ____ days after treatment initiation |
| --- |

| Name:________________________D.o.b.:____________ | **NH form – IV ANTIBIOTICS p5** |
| --- | --- |

*Decision-making process – To be filled out by the nursing home physician*

| 1. Was there ever any doubt whether intravenous treatment was right for this patient?  Yes  No  If yes, were any of the following the reasons?   Doubt whether the treatment was for the patient’s best   Doubt whether the patient actually wanted intravenous treatment   Uncertainty about what kind of treatment next of kin wanted the patient to have   Doubt whether the treatment would bring the desired effect   Disagreement within the treatment team regarding the treatment   Other ________________________________________________________________  2. Was the treatment discussed with nursing home staff who knew the patient?  Yes  No  Don’t know  If no, why not? ________________________________________________________________  3. Was the treatment discussed with the patient before initiation?  Yes  No  Don’t know  If no, why not? ________________________________________________________________  5. Was the treatment discussed with next of kin before initiation?  Yes  No  Don’t know  If no, why not? ________________________________________________________________  6. Was the patient’s competence to consent assessed at the hospital before treatment?   Yes  No  Don’t know  If yes, was the patient competent?  Yes  No  If no, why was competence not assessed (mark all that apply)?   The patient was obviously competent   The patient was obviously not competent   We did not have time for an assessment   Uncertainty about how to assess competence   Other _________________________________________________________________  7. Have there been conversations with the patient and next of kin regarding the patient’s wishes and values regarding life-prolonging treatment, or what to do if the patient’s health suddenly deteriorates?   Yes  No  Don’t know |
| --- |
