# Supplementary material for: Involvement in decisions about intravenous treatment for nursing home patients: nursing homes versus hospital wards
Source: BMC Med Ethics. 2018 May 8;19:34. doi: 10.1186/s12910-018-0258-5 (PMC5941318; doi:10.1186/s12910-018-0258-5)
Supplement: Supplementary file 5 — Research form. Form for patients treated with iv fluids in nursing homes. (DOC 62 kb) [file 12910_2018_258_MOESM5_ESM.doc]

| Name:________________________D.o.b.:____________ | **NH form – IV FLUID p1** |
| --- | --- |

| **Reason for dehydration** | Resists fluid intake  Diarrhea  Vomiting  Fever/infection  Weeping wounds Medication  Heath wave  Other  Unsure |  No  Yes   No  Yes   No  Yes   No  Yes ___________________________________   No  Yes   No  Yes _________________________________   No  Yes   No  Yes ________________________________   No  Yes ________________________________ |
| --- | --- | --- |

Intravenous fluid treatment

| Date | Time of initiation | Type of fluid | Daily dose  (ml) | Doserage | Day  1 (x) | Day  2 (x) | Day  3 (x) | Day  4 (x) | Day  5 (x) | Day  6 (x) | Day  7 (x) |
| --- | --- | --- | --- | --- | --- | --- | --- | --- | --- | --- | --- |
|  |  |  |  |  |  |  |  |  |  |  |  |
|  |  |  |  |  |  |  |  |  |  |  |  |
|  |  |  |  |  |  |  |  |  |  |  |  |

Cessation of medication: ____________________________________________________________________________

Other treatment initiated:__________________________________________________________________________

_________________________________________________________________________________________________

Clinical status day 1

| BP ____/____ Pulse _____ Temp ___.__ Respiration frequency____ CRP ____ (if taken)  Consciousness  Awake  Somnolence  Unconscious  *Fill out for those patients who had aberrant results on day 1* |
| --- |

|  Confusion Assessment Method (CAM) filled out |
| --- |

Important notes ___________________________________________________________________________________

_________________________________________________________________________________________________

Clinical status day 3

| BP ____/____ Pulse _____ Temp ___.__ Respiration frequency____ CRP ____ (if taken)  Consciousness  Awake  Somnolence  Unconscious  *Fill out for those patients who had aberrant results on day 1* |
| --- |

|  Confusion Assessment Method (CAM) filled out |
| --- |

Important notes ______________________________________________________________

_________________________________________________________________________________________________

| Name:________________________D.o.b.:____________ | **NH form – IV FLUID p2** |
| --- | --- |

Clinical status day 5

| BP ____/____ Pulse _____ Temp ___.__ Respiration frequency____ CRP ____ (if taken)  Consciousness  Awake  Somnolence  Unconscious  *Fill out for those patients who had aberrant results on day 3* |
| --- |

|  Confusion Assessment Method (CAM) filled out |
| --- |

Important notes ___________________________________________________________________________________

_________________________________________________________________________________________________

*Decision-making process – To be filled out by the nursing home physician*

| 1. Was there ever any doubt whether intravenous treatment was right for this patient?  Yes  No  If yes, were any of the following the reasons?   Doubt whether the treatment was for the patient’s best   Doubt whether the patient actually wanted intravenous treatment   Uncertainty about what kind of treatment next of kin wanted the patient to have   Doubt whether the treatment would bring the desired effect   Disagreement within the treatment team regarding the treatment   Other ________________________________________________________________  2. Was the treatment discussed with nursing home staff who knew the patient?  Yes  No  Don’t know  If no, why not? ________________________________________________________________  3. Was the treatment discussed with the patient before initiation?  Yes  No  Don’t know  If no, why not? ________________________________________________________________  5. Was the treatment discussed with next of kin before initiation?  Yes  No  Don’t know  If no, why not? ________________________________________________________________  6. Was the patient’s competence to consent assessed at the hospital before treatment?   Yes  No  Don’t know  If yes, was the patient competent?  Yes  No  If no, why was competence not assessed (mark all that apply)?   The patient was obviously competent   The patient was obviously not competent   We did not have time for an assessment   Uncertainty about how to assess competence   Other _________________________________________________________________  7. Have there been conversations with the patient and next of kin regarding the patient’s wishes and values regarding life-prolonging treatment, or what to do if the patient’s health suddenly deteriorates?   Yes  No  Don’t know |
| --- |

| Name:________________________D.o.b.:____________ | **NH form – IV FLUID p3** |
| --- | --- |

Summary 14 days after debut of current illness

| **Course of illness**  ___ days of intravenous fluid  ___ days before clinically well  Back to normal condition?  Yes  No  Do not know patient’s normal condition   Dead ___ days after treatment initiation | | |
| --- | --- | --- |
| **Complications**  Bed sore  Fall with injury  Delirium  Hospital infection  Other |  No Yes:   No Yes:   No Yes:   No Yes:   No Yes: | ________________________________________  ________________________________________  ___ days  ________________________________________  ________________________________________ |
| **Intravenous treatment**  Complications from intravenous treatment (infection in vein, hematoma, failure in equipment etc)   No  Yes:_______________________________________________________________________________  Challenges/disadvantages of treating this patient in the nursing home: _________________________________  _________________________________________________________________________________________  _________________________________________________________________________________________  Advantages to treating this patient in the nursing home: ____________________________________________  _________________________________________________________________________________________  _________________________________________________________________________________________ | | |

Check list 14 days after debut of current illness

|  Consent form attached (patient’s signature of participation in the study)   Barthel ADL-Index filled out – for situation 30 days after debut of illness   Copy of current medication list attached   Patient is discharged to the home. Call: Lisbeth Østby, 91820728   Patient is dead ____ days after treatment initiation |
| --- |
